# Supplementary material for: The Small RNA Universe of Capitella teleta
Source: Front Mol Biosci. 2022 Feb 25;9:802814. doi: 10.3389/fmolb.2022.802814 (PMC8915122; doi:10.3389/fmolb.2022.802814)
Supplement: Supplementary file 1 [file DataSheet1.ZIP › Supplement/known/CAPTEscaffold_135_11088.pdf]

Provisional ID	:	CAPTEscaffold_135_11088
Score total	:	560271.4
Score for star read(s)	:	3.9
Score for read counts	:	560261.2
Score for mfe	:	1.7
Score for randfold	:	1.6
Score for cons. seed	:	3
Total read count	:	1098940
Mature read count	:	1098505
Loop read count	:	0
Star read count	:	435

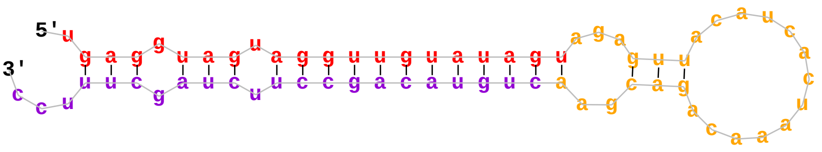

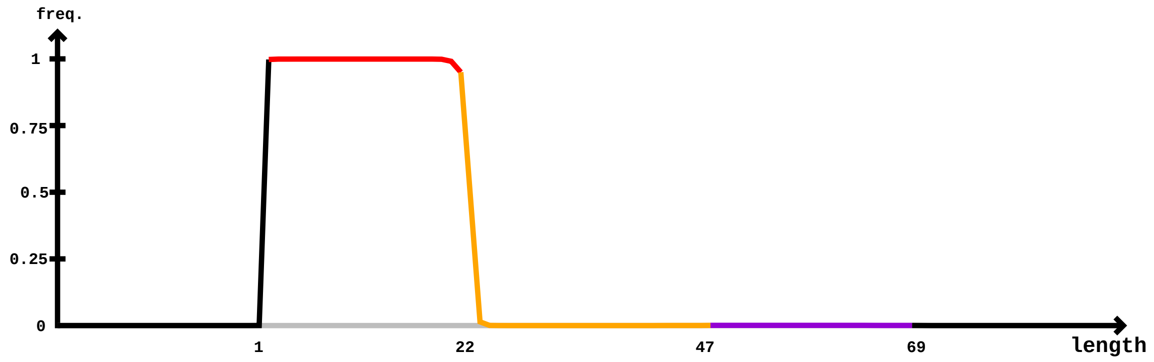

Mature Star

```
5'- cccccuggcuuaccuccaguugagguaguagguuguauaguagaguuacaucacuaaacagacgaacuguacagccuucuagcuuuccugggguacagcacugcugcau   -3'   obs
    cccccuggcuuaccuccaguugagguaguagguuguauaguagaguuacaucacuaaacagacgaacuguacagccuucuagcuuuccugggguacagcacugcugcau         exp
    cccccuggcuuaccuccaguugagguaguagguuguauaguagaguuacaucacuaaacagacgaacuguacagccuucuagcuuuccugggguacagcacugcugcau         known
    .......(((((((.((((..(((.(((.((((((((((((...(((.............)))..)))))))))))).))).)))..)))))))).)))..........   reads   mm   sample

    cccccuggcuuaccuccagu.........................................................................................   10      0    seq
    .................aguugagguaguagguuguau.......................................................................   12      0    seq
    .................agAugagguaguagguuguauagu....................................................................   2       1    seq
    ...................Cugagguaguagguugua........................................................................   1       1    seq
    ...................Cugagguaguagguuguauag.....................................................................   1       1    seq
    ...................Cugagguaguagguuguauagu....................................................................   120     1    seq
    ...................uuAagguaguagguuguauagu....................................................................   1       1    seq
    ...................Augagguaguagguuguauagu....................................................................   22      1    seq
    ...................uugagguaguagguuguauagu....................................................................   3       0    seq
    ...................Cugagguaguagguuguauagua...................................................................   21      1    seq
    ....................ugagguagAagguuguau.......................................................................   1       1    seq
    ....................ugagguaguaAguuguau.......................................................................   1       1    seq
    ....................Agagguaguagguuguau.......................................................................   6       1    seq
    ....................ugagguaguagguuguUu.......................................................................   5       1    seq
    ....................ugagguaguagUuuguau.......................................................................   1       1    seq
    ....................ugaggAaguagguuguau.......................................................................   1       1    seq
    ....................Ggagguaguagguuguau.......................................................................   2       1    seq
    ....................ugagguaguagguuguau.......................................................................   751     0    seq
    ....................ugagguaguagguuguaA.......................................................................   43      1    seq
    ....................ugagguaguGgguuguaua......................................................................   6       1    seq
    ....................ugagguagGagguuguaua......................................................................   3       1    seq
    ....................ugagguaguaggCuguaua......................................................................   1       1    seq
    ....................ugagguaCuagguuguaua......................................................................   1       1    seq
    ....................ugagguaguagguugAaua......................................................................   4       1    seq
    ....................ugagguaguagguuguUua......................................................................   2       1    seq
    ....................Ggagguaguagguuguaua......................................................................   7       1    seq
    ....................ugagguaguagguuguaua......................................................................   8120    0    seq
    ....................ugaggAaguagguuguaua......................................................................   1       1    seq
    ....................ugagguaguagguugCaua......................................................................   2       1    seq
    ....................ugagguaguagguuguaAa......................................................................   22      1    seq
    ....................uAagguaguagguuguaua......................................................................   6       1    seq
```

```
                    Mature                                        Star
cccccuggcuuaccuccaguugagguaguagguuguauaguagaguuacaucacuaaacagacgaacuguacagccuucuagcuuuccugggguacagcacugcugcau
....................ugagguaguagguuCuaua......................................................................   1       1       seq
....................ugaggGaguagguuguaua......................................................................   1       1       seq
....................ugagguaguaggAuguaua......................................................................   3       1       seq
....................ugagguaguagguAguaua......................................................................   2       1       seq
....................ugagguaguaAguuguaua......................................................................   4       1       seq
....................ugagguaUuagguuguaua......................................................................   1       1       seq
....................ugGgguaguagguuguaua......................................................................   1       1       seq
....................ugagguaguagAuuguaua......................................................................   3       1       seq
....................ugagguaguUgguuguaua......................................................................   1       1       seq
....................ugaAguaguagguuguaua......................................................................   2       1       seq
....................ugagguaguaUguuguaua......................................................................   3       1       seq
....................uUagguaguagguuguaua......................................................................   1       1       seq
....................Agagguaguagguuguaua......................................................................   32      1       seq
....................ugagguaguagguuguauU......................................................................   96      1       seq
....................ugagguaguagguuguauG......................................................................   9       1       seq
....................ugagguaguagguCguaua......................................................................   2       1       seq
....................Ngagguaguagguuguaua......................................................................   1       1       seq
....................ugagguGguagguuguaua......................................................................   2       1       seq
....................ugagguaAuagguuguaua......................................................................   1       1       seq
....................ugagguaguagguuguGua......................................................................   24      1       seq
....................ugagguaguagguuguaGag.....................................................................   2       1       seq
....................ugagguaguagguuguaCag.....................................................................   4       1       seq
....................ugagAuaguagguuguauag.....................................................................   9       1       seq
....................ugUgguaguagguuguauag.....................................................................   3       1       seq
....................ugagguaguagguuguauag.....................................................................   44222   0       seq
....................uCagguaguagguuguauag.....................................................................   6       1       seq
....................ugagguagCagguuguauag.....................................................................   4       1       seq
....................ugagguaguagguuguauaU.....................................................................   42      1       seq
....................ugagguagAagguuguauag.....................................................................   5       1       seq
....................ugagguaCuagguuguauag.....................................................................   4       1       seq
....................uUagguaguagguuguauag.....................................................................   5       1       seq
....................Cgagguaguagguuguauag.....................................................................   7       1       seq
....................ugagguaguagguuAuauag.....................................................................   4       1       seq
....................ugagguaUuagguuguauag.....................................................................   2       1       seq
....................ugagUuaguagguuguauag.....................................................................   1       1       seq
....................ugaggCaguagguuguauag.....................................................................   4       1       seq
....................ugagguUguagguuguauag.....................................................................   4       1       seq
....................ugagguaguagguAguauag.....................................................................   3       1       seq
....................ugagguaguaggGuguauag.....................................................................   5       1       seq
....................ugagguaguagguuguaAag.....................................................................   8       1       seq
....................uAagguaguagguuguauag.....................................................................   26      1       seq
....................ugagguaAuagguuguauag.....................................................................   2       1       seq
....................ugGgguaguagguuguauag.....................................................................   4       1       seq
....................ugagguaguagguCguauag.....................................................................   12      1       seq
....................Ggagguaguagguuguauag.....................................................................   61      1       seq
....................ugagguGguagguuguauag.....................................................................   11      1       seq
....................ugagguaguCgguuguauag.....................................................................   1       1       seq
....................ugagCuaguagguuguauag.....................................................................   4       1       seq
....................ugagguaguagguugAauag.....................................................................   15      1       seq
....................ugaggGaguagguuguauag.....................................................................   1       1       seq
....................ugagguaguagguugCauag.....................................................................   3       1       seq
....................ugagguagGagguuguauag.....................................................................   4       1       seq
....................ugaCguaguagguuguauag.....................................................................   4       1       seq
....................ugagguaguagguugGauag.....................................................................   4       1       seq
....................ugaUguaguagguuguauag.....................................................................   3       1       seq
....................ugagguaguagAuuguauag.....................................................................   1       1       seq
....................ugaAguaguagguuguauag.....................................................................   12      1       seq
....................ugagguaguaCguuguauag.....................................................................   2       1       seq
....................ugagguaguagguuUuauag.....................................................................   4       1       seq
....................ugagguaguagUuuguauag.....................................................................   2       1       seq
....................ugagguaguaAguuguauag.....................................................................   8       1       seq
....................ugagguaguagguGguauag.....................................................................   4       1       seq
....................ugagguaguaggCuguauag.....................................................................   21      1       seq
....................ugagguaguaUguuguauag.....................................................................   4       1       seq
....................Agagguaguagguuguauag.....................................................................   163     1       seq
....................ugagguaguGgguuguauag.....................................................................   40      1       seq
....................ugagguaguagguuguauaC.....................................................................   2       1       seq
....................ugagguaguaggAuguauag.....................................................................   7       1       seq
....................ugagguaguagguuguUuag.....................................................................   5       1       seq
....................ugagguaguagguuguauGg.....................................................................   4       1       seq
```

Mature Star

```
cccccuggcuuaccuccaguugagguaguagguuguauaguagaguuacaucacuaaacagacgaacuguacagccuucuagcuuuccugggguacagcacugcugcau
....................Ngagguaguagguuguauag.....................................................................   17      1   seq
....................ugagguaguagguuguauaA.....................................................................   99      1   seq
....................ugagguaguUgguuguauag.....................................................................   4       1   seq
....................ugaggAaguagguuguauag.....................................................................   17      1   seq
....................ugagguaguagguuguGuag.....................................................................   5       1   seq
....................ugagguaguagguuguauUg.....................................................................   5       1   seq
....................ugagguagNagguuguauagu....................................................................   1       1   seq
....................ugaggNaguagguuguauagu....................................................................   11      1   seq
....................ugagguaguagCuuguauagu....................................................................   21      1   seq
....................ugagguaguaAguuguauagu....................................................................   246     1   seq
....................ugagguaUuagguuguauagu....................................................................   30      1   seq
....................ugagguaguagguuguauagG...................................................................   238     1   seq
....................ugagguaguagguCguauagu....................................................................   118     1   seq
....................ugUgguaguagguuguauagu....................................................................   43      1   seq
....................ugagguagCagguuguauagu....................................................................   83      1   seq
....................ugagguaguagguuguaAagu....................................................................   164     1   seq
....................ugagguaguagguugAauagu....................................................................   358     1   seq
....................ugagguaguagguuguUuagu....................................................................   91      1   seq
....................uUagguaguagguuguauagu....................................................................   100     1   seq
....................ugagguaguagUuuguauagu....................................................................   20      1   seq
....................ugaggAaguagguuguauagu....................................................................   222     1   seq
....................ugagguaguagAuuguauagu....................................................................   147     1   seq
....................ugagguaguagguuguauaAu....................................................................   45      1   seq
....................ugagguaguagguuguaGagu....................................................................   64      1   seq
....................ugagguaguagguuguaCagu....................................................................   74      1   seq
....................ugagguaguaggAuguauagu....................................................................   153     1   seq
....................ugaAguaguagguuguauagu....................................................................   226     1   seq
....................ugagguaAuagguuguauagu....................................................................   49      1   seq
....................ugagguagGagguuguauagu....................................................................   74      1   seq
....................ugagguaguagguuguauagA...................................................................   3679    1   seq
....................ugaUguaguagguuguauagu....................................................................   30      1   seq
....................uNagguaguagguuguauagu....................................................................   25      1   seq
....................ugagguaguagguGguauagu....................................................................   95      1   seq
....................ugagguaguagguuguauUgu....................................................................   55      1   seq
....................ugagguaguagguuguauGgu....................................................................   68      1   seq
....................ugagguaguagguAguauagu....................................................................   103     1   seq
....................ugagCuaguagguuguauagu....................................................................   27      1   seq
....................ugagguaguUgguuguauagu....................................................................   63      1   seq
....................ugGgguaguagguuguauagu....................................................................   59      1   seq
....................ugagguGguagguuguauagu....................................................................   143     1   seq
....................ugagUuaguagguuguauagu....................................................................   30      1   seq
....................ugagguaguagguuguauaCu....................................................................   18      1   seq
....................ugagguaguGgguuguauagu....................................................................   907     1   seq
....................Ggagguaguagguuguauagu....................................................................   781     1   seq
....................ugagguUguagguuguauagu....................................................................   47      1   seq
....................ugagguaguagguuAuauagu....................................................................   93      1   seq
....................ugNgguaguagguuguauagu....................................................................   5       1   seq
....................ugagguaguCgguuguauagu....................................................................   4       1   seq
....................ugagAuaguagguuguauagu....................................................................   81      1   seq
....................ugaCguaguagguuguauagu....................................................................   21      1   seq
....................ugagguaguagguuguNuagu....................................................................   3       1   seq
....................Cgagguaguagguuguauagu....................................................................   130     1   seq
....................ugagguaguaggGuguauagu....................................................................   79      1   seq
....................ugagguaguagguuguauaNu....................................................................   1       1   seq
....................Agagguaguagguuguauagu....................................................................   2634    1   seq
....................ugagguNguagguuguauagu....................................................................   1       1   seq
....................ugagguaguagguuUuauagu....................................................................   41      1   seq
....................ugagguaguaUguuguauagu....................................................................   28      1   seq
....................ugagguaguagguuguGuagu....................................................................   95      1   seq
....................Ngagguaguagguuguauagu....................................................................   219     1   seq
....................ugagguaguaCguuguauagu....................................................................   17      1   seq
....................ugagguaCuagguuguauagu....................................................................   40      1   seq
....................ugagguaguagguugCauagu....................................................................   79      1   seq
....................ugagNuaguagguuguauagu....................................................................   1       1   seq
....................ugagguaguagguuguauagu....................................................................   712598  0   seq
....................ugagguaguagguuCuauagu....................................................................   20      1   seq
....................ugagguaguagguuguauagC...................................................................   59      1   seq
....................ugaggCaguagguuguauagu....................................................................   68      1   seq
....................uCagguaguagguuguauagu....................................................................   108     1   seq
....................ugagguagAagguuguauagu....................................................................   192     1   seq
```

Mature   Star

```
cccccuggcuuaccuccaguugagguaguagguuguauaguagaguuacaucacuaaacagacgaacuguacagccuucuagcuuuccugggguacagcacugcugcau
....................ugagguaguaggCuguauagu....................................................................   90      1   seq
....................ugagguCguagguuguauagu....................................................................   4       1   seq
....................uAagguaguagguuguauagu....................................................................   657     1   seq
....................ugagguaguNgguuguauagu....................................................................   3       1   seq
....................ugagguaguagguuguauaUu....................................................................   25      1   seq
....................ugCgguaguagguuguauagu....................................................................   10      1   seq
....................ugaggGaguagguuguauagu....................................................................   69      1   seq
....................ugagguaguagguugGauagu....................................................................   51      1   seq
....................ugagguaguagguuguauCgu....................................................................   8       1   seq
....................ugagguaguagguugCauagua...................................................................   85      1   seq
....................ugagguaguagguuguGuagua...................................................................   36      1   seq
....................ugagguaguagguuguauaUua...................................................................   20      1   seq
....................ugagguaguaCguuguauagua...................................................................   4       1   seq
....................ugagguaguagguuguauaAua...................................................................   8       1   seq
....................ugagguaguaUguuguauagua...................................................................   13      1   seq
....................ugagguaguagguuAuauagua...................................................................   30      1   seq
....................uCagguaguagguuguauagua...................................................................   30      1   seq
....................uUagguaguagguuguauagua...................................................................   29      1   seq
....................ugagguaguagguCguauagua...................................................................   41      1   seq
....................ugagguaguagguuguauagNa...................................................................   3       1   seq
....................uAagguaguagguuguauagua...................................................................   256     1   seq
....................ugagUuaguagguuguauagua...................................................................   16      1   seq
....................ugagguaguagguuguauCgua...................................................................   3       1   seq
....................ugagguaguaggGuguauagua...................................................................   60      1   seq
....................ugagguaguagguAguauagua...................................................................   43      1   seq
....................ugagCuaguagguuguauagua...................................................................   11      1   seq
....................ugagguaguagguugGauagua...................................................................   18      1   seq
....................ugagguaguagguGguauagua...................................................................   9       1   seq
....................ugagguaguagguuguauGgua...................................................................   32      1   seq
....................ugagguaguaAguuguauagua...................................................................   75      1   seq
....................ugagguaguagguuguauaCua...................................................................   5       1   seq
....................ugagguaguagguuguaAagua...................................................................   57      1   seq
....................uNagguaguagguuguauagua...................................................................   2       1   seq
....................ugagguaguagguuguauagGa...................................................................   21      1   seq
....................ugagguaUuagguuguauagua...................................................................   12      1   seq
....................ugagguaguagguuguCuagua...................................................................   1       1   seq
....................ugagguaguagAuuguauagua...................................................................   20      1   seq
....................ugagguaguagguuguauaguU...................................................................   2542    1   seq
....................ugaggNaguagguuguauagua...................................................................   6       1   seq
....................ugagguaguagguuguauaNua...................................................................   1       1   seq
....................ugGgguaguagguuguauagua...................................................................   19      1   seq
....................ugagguaCuagguuguauagua...................................................................   19      1   seq
....................ugUgguaguagguuguauagua...................................................................   23      1   seq
....................ugagguaguaggAuguauagua...................................................................   68      1   seq
....................ugaCguaguagguuguauagua...................................................................   11      1   seq
....................ugNgguaguagguuguauagua...................................................................   1       1   seq
....................ugagguGguagguuguauagua...................................................................   56      1   seq
....................ugagAuaguagguuguauagua...................................................................   35      1   seq
....................ugagguagCagguuguauagua...................................................................   36      1   seq
....................Ngagguaguagguuguauagua...................................................................   102     1   seq
....................ugagguagNagguuguauagua...................................................................   1       1   seq
....................ugagguaguagguuguaGagua...................................................................   32      1   seq
....................ugagguaguagguuguauUgua...................................................................   20      1   seq
....................ugagguaguagguuguauagua...................................................................   294367  0   seq
....................ugagguaguaggCuguauagua...................................................................   23      1   seq
....................ugagguaguagguUuauagua....................................................................   17      1   seq
....................ugagguaguagguuguaCagua...................................................................   36      1   seq
....................ugagguagAagguuguauagua...................................................................   64      1   seq
....................ugaAguaguagguuguauagua...................................................................   101     1   seq
....................ugaggAaguagguuguauagua...................................................................   86      1   seq
....................ugagguagGagguuguauagua...................................................................   40      1   seq
....................ugagguaguGgguuguauagua...................................................................   223     1   seq
....................ugagguaguagguuguauagCa...................................................................   24      1   seq
....................ugaUguaguagguuguauagua...................................................................   9       1   seq
....................ugagguCguagguuguauagua...................................................................   2       1   seq
....................ugagguaguagguuguUuagua...................................................................   42      1   seq
....................Cgagguaguagguuguauagua...................................................................   45      1   seq
....................ugagguaguaNguuguauagua...................................................................   1       1   seq
....................ugagguaguagguugNauagua...................................................................   1       1   seq
....................Ggagguaguagguuguauagua...................................................................   340     1   seq
```

Mature **Star**

```
cccccuggcuuaccuccaguugagguaguagguuguauaguagaguuacaucacuaaacagacgaacuguacagccuucuagcuuuccugggguacagcacugcugcau
....................ugagguaguagguuguauagAa...................................................................  203   1  seq
....................Agagguaguagguuguauagua...................................................................  1022  1  seq
....................ugagguaguagUuuguauagua...................................................................  8     1  seq
....................ugagguaAuagguuguauagua...................................................................  16    1  seq
....................ugagguaguagguuCuauagua...................................................................  13    1  seq
....................ugagguaguagCuuguauagua...................................................................  8     1  seq
....................ugagguaguagguugAauagua...................................................................  128   1  seq
....................ugagguUguagguuguauagua...................................................................  24    1  seq
....................ugagguaguUgguuguauagua...................................................................  40    1  seq
....................ugaggCaguagguuguauagua...................................................................  30    1  seq
....................ugagguaguCgguuguauagua...................................................................  1     1  seq
....................ugagguaguagguuguauaguC...................................................................  87    1  seq
....................ugagguaguagguuguauaguG...................................................................  134   1  seq
....................ugaggGaguagguuguauagua...................................................................  21    1  seq
....................ugagguaguagguuguauaguag..................................................................  22    0  seq
....................ugagguaguagguuguauaguaC..................................................................  440   1  seq
....................ugagguaguagguuguauaguaA..................................................................  5821  1  seq
....................ugagguaguagguuguauaguaU..................................................................  8417  1  seq
....................ugagguaguagguuguauaguUg..................................................................  13    1  seq
....................ugaggGaguagguuguauaguag..................................................................  1     1  seq
....................ugagguaguagguuguauaguaga.................................................................  1     0  seq
....................ugagguaguagguuguauaguaAa.................................................................  435   1  seq
....................ugagguaguagguuguauaguaUa.................................................................  36    1  seq
....................ugagguaguagguuguauaguagaAu...............................................................  1     1  seq
....................ugagguaguagguuguauaguagagu...............................................................  1     0  seq
....................ugagguaguagguuguauaguagaguu..............................................................  2     0  seq
.....................gagguaguagguuguaua......................................................................  4     0  seq
.....................Uagguaguagguuguauag.....................................................................  2     1  seq
.....................gUgguaguagguuguauag.....................................................................  1     1  seq
.....................gagguagGagguuguauag.....................................................................  1     1  seq
.....................gagguaguagguuguauag.....................................................................  33    0  seq
.....................gagguaguagguuguauUgu....................................................................  2     1  seq
.....................gagguaguagguuguaAagu....................................................................  2     1  seq
.....................Uagguaguagguuguauagu....................................................................  20    1  seq
.....................gagguaguagguAguauagu....................................................................  1     1  seq
.....................gagguaguagguugGauagu....................................................................  1     1  seq
.....................gagguaguagguugAauagu....................................................................  1     1  seq
.....................gagguaguaUguuguauagu....................................................................  1     1  seq
.....................gagguaguagguuguaCagu....................................................................  1     1  seq
.....................gagguaguagguuguauagu....................................................................  962   0  seq
.....................gagguaguagguuguauagA....................................................................  1     1  seq
.....................gagguaguaggAuguauagu....................................................................  1     1  seq
.....................gGgguaguagguuguauagu....................................................................  3     1  seq
.....................gagguagGagguuguauagu....................................................................  4     1  seq
.....................gaggGaguagguuguauagu....................................................................  1     1  seq
.....................Uagguaguagguuguauagua...................................................................  7     1  seq
.....................gagguaAuagguuguauagua...................................................................  1     1  seq
.....................gagguaguagguuguauagua...................................................................  502   0  seq
.....................gagguaguaggGuguauagua...................................................................  1     1  seq
.....................gagguaguGgguuguauagua...................................................................  2     1  seq
.....................gagguaguaggAuguauagua...................................................................  2     1  seq
.....................gagguaUuagguuguauagua...................................................................  1     1  seq
.....................gaggAaguagguuguauagua...................................................................  1     1  seq
.....................gagguaguagguuguauaguaA..................................................................  2     1  seq
.....................gagguaguagguuguauaguaU..................................................................  20    1  seq
.....................gagguaguagguuguauaguagUg................................................................  1     1  seq
.....................gagguaguagguuguauUguagag................................................................  1     1  seq
.....................gagguaguagguuguauaguagag................................................................  86    0  seq
......................agguaguagguuguauagu....................................................................  9     0  seq
......................Ugguaguagguuguauagu....................................................................  6     1  seq
......................Ugguaguagguuguauagua...................................................................  4     1  seq
......................aUguaguagguuguauagua...................................................................  1     1  seq
......................agguaguagguuguauagua...................................................................  1     0  seq
.......................gguaguaggAuguauagu....................................................................  1     1  seq
.......................Uguaguagguuguauagu....................................................................  1     1  seq
.......................gguaguagguuguauagu....................................................................  9     0  seq
.......................gguaguagguuguauagua...................................................................  7     0  seq
.......................gguaguagguuguauaUua...................................................................  1     1  seq
........................guaguagguuguauaguagagu...............................................................  2     0  seq
.........................uaguagguuguauaguaA..................................................................  22    1  seq
```

Mature **Star**

```
cccccuggcuuaccuccaguugagguaguagguuguauaguagaguuacaucacuaaacagacgaacuguacagccuucuagcuuuccugggguacagcacugcugcau
.........................uaguagguuguauaguagagu...............................................................	4	0	seq
.........................uaguagguuguauaguagaguu..............................................................	22	0	seq
.........................uaguagguuguauaguagaguua.............................................................	2	0	seq
..........................................................cagacgaacuguacagccuucuagcuuucc.....................	1	0	seq
.............................................................acgaacuCuacagccuucuagc..........................	1	1	seq
.............................................................acgaacuguacagccuucuagc..........................	209	0	seq
.............................................................acgaacuguacagcAuucuagc..........................	1	1	seq
..............................................................cgaacuguacagccuucuagc..........................	23	0	seq
..............................................................cAaacuguacagccuucuagcu.........................	1	1	seq
..............................................................cgaacuguacagccuucuagcu.........................	86	0	seq
..............................................................cgaacuguacagccuucuagcuuuc......................	1	0	seq
...............................................................gaacuguacagccuucuagcu.........................	1	0	seq
..................................................................cuguacagccuucuagcu.........................	1	0	seq
..................................................................cuguacagcAuucuagcuuuc......................	1	1	seq
..................................................................cuguacagccuucuagcuuuU......................	2	1	seq
..................................................................cuguacagccuucuagcuuuc......................	7	0	seq
..................................................................Uuguacagccuucuagcuuucc.....................	1	1	seq
..................................................................cuguacagccuucuagcuuucc.....................	382	0	seq
..................................................................cuguacagAcuucuagcuuucc.....................	1	1	seq
..................................................................Auguacagccuucuagcuuucc.....................	1	1	seq
..................................................................cuguacagccuucuagcuuuccA....................	18	1	seq
..................................................................cuguacagccuucuagcuuuccu....................	5	0	seq
...................................................................uguacagccuucuagcuuucc.....................	1	0	seq
...................................................................uguacagccuucuagcuuuccA....................	4	1	seq
...................................................................uguacagccuucuagcuuuccu....................	10	0	seq
...................................................................uguacagccuucuagcuuuccuU...................	1	1	seq
```
